# *Notes from the Field:* Neurosyphilis, Ocular Syphilis, and Otic Syphilis — Chicago, January–October 2023

**DOI:** 10.15585/mmwr.mm7408a3

**Published:** 2025-03-13

**Authors:** Amy Nham, Taylor Holly, John Flores, David Kern, Irina Tabidze

**Affiliations:** ^1^Epidemic Intelligence Service, CDC; ^2^Chicago Department of Public Health, Chicago, Illinois; ^3^University of Chicago Medicine, Chicago, Illinois.

**Summary**

**What is already known about this topic?**

High rates of syphilis and HIV coinfection occur among gay, bisexual, and other men who have sex with men (MSM). Neurosyphilis, ocular syphilis, and otic syphilis (NOO syphilis) can occur at any syphilis stage with or without HIV coinfection.

**What is added by this report?**

During January 1–October 31, 2023, 40 NOO syphilis cases were reported in Chicago, 67.5% of which occurred in persons without HIV infection compared with 43.8% in 2019. Among 33 (82.5%) NOO syphilis patients whose sex and that of their sexual partners were reported, 18 (54.5%) were not MSM compared with four of 15 patients (26.7%) in 2019.

**What are the implications for public health practice?**

Clinicians should consider NOO syphilis even in persons who do not have HIV and who are not MSM.

The incidence of syphilis, a sexually transmitted infection caused by the bacterium *Treponema pallidum,* has been increasing in the United States since 2001.* Neurosyphilis, ocular syphilis, and otic syphilis (NOO syphilis), caused by *T. pallidum* invading the central nervous system, eyes, and ears, respectively, can occur in the primary, secondary, latent, or tertiary^†^ stages of syphilis. NOO syphilis manifestations are often debilitating and can include meningitis, stroke, motor or sensory deficits, blindness, and hearing loss.

Data from the early to mid-2000s suggested that NOO syphilis was more commonly identified among gay, bisexual, and other men who have sex with men (MSM) and persons with HIV, likely reflecting the syphilis trends at the time these data were collected (*1*–*3*). Data collected from Chicago in 2019 showed that 11 of 16 NOO syphilis cases were diagnosed in MSM, and nine cases in persons with HIV. In 2023, health care providers in Chicago reported an increase in NOO syphilis among heterosexual persons without HIV. To assess whether this increase was consistent citywide, this analysis characterized NOO syphilis cases stratified by HIV status to identify factors associated with diagnosis in Chicago during January 1–October 31, 2023.

## Investigation and Outcomes

### Data Collection and Analyses

Surveillance data reported to the Chicago Department of Public Health (CDPH) were analyzed to describe NOO syphilis cases diagnosed in 2023. CDPH queried the Chicago Health Information Management System (CHIMS) to identify potential NOO syphilis cases based on 1) cerebrospinal fluid (CSF) test results indicative of neurosyphilis, including reactive CSF Venereal Disease Research Laboratory (VDRL), elevated CSF protein, and elevated CSF leukocyte count, 2) treatment with intravenous penicillin, or 3) any documented NOO syphilis sign or symptom in a Chicago resident during January 1–October 31, 2023. (*4*). CDPH abstracted clinical information from medical records to identify cases that met the 2018 Council of State and Territorial Epidemiologists’ verified, likely, or possible case definitions for NOO syphilis (*4*). CDPH matched cases identified from CHIMS to the Enhanced HIV/AIDS Reporting System to obtain data on HIV status and treatment (*5*). Data from the National Electronic Telecommunications System for Surveillance were used to obtain 2019 case data for comparison, excluding COVID-19 pandemic years in which cases were likely underreported. This activity was reviewed by CDC, deemed not research, and was conducted consistent with applicable federal law and CDC policy.^§^

### Outcomes

During January 1–October 31, 2023, a total of 2,611 cases of syphilis (all stages) were reported to CDPH; 689 (26.4%) were among persons with HIV, and 521 (20.0%) were among MSM. Among all 2,611 cases, 40 (1.5%) NOO syphilis cases were reported, including 14 in patients who had more than one type of NOO syphilis (Table). Overall, 28 (70.0%) neurosyphilis cases (19 verified, four likely, and five possible), 24 (60.0%) ocular syphilis cases (17 likely and seven possible), and two (5.0%) otic syphilis cases (both possible) were reported. Patients ranged in age from 23 to 82 years (median = 46.5 years), 29 (72.5%) were male, and 26 (65.0%) were non-Hispanic Black or African American persons. Twenty-seven (67.5%) cases occurred among persons who did not have HIV (compared with less than one half [seven of 16] in 2019), and among 33 patients whose sex and that of their sexual partners were documented, 18 (54.5%) were not MSM (compared with 26.7% [four of 15] in 2019).

**TABLE:** Demographic and clinical presentation characteristics of persons with neurosyphilis, ocular syphilis, or otic syphilis, by HIV status* — Chicago, 2019 and January 1–October 31, 2023

Characteristic	No. (column %)			
	2019^†^	January 1–October 31, 2023		
	Total N = 16^†^	Total N = 40	Persons with HIV n = 13	Persons without HIV n = 27
**Type of syphilis**				
Neurosyphilis	**—**	**28 (70.0)**	—	—
Ocular syphilis	**—**	**24 (60.0)**	—	—
Otic syphilis	**—**	**2 (5.0)**	—	—
More than one type of NOO syphilis	**—**	**14 (35.0)**	—	—
**Age group, yrs**				
20–29	**6 (37.5)**	**8 (20.0)**	2 (15.4)	6 (22.2)
30–39	**6 (37.5)**	**7 (17.5)**	3 (23.1)	4 (14.8)
40–49	**3 (18.8)**	**6 (15.0)**	4 (30.8)	2 (7.4)
50–59	**1 (6.3)**	**8 (20.0)**	2 (15.4)	6 (22.2)
≥60	**0 (—)**	**11 (27.5)**	2 (15.4)	9 (33.3)
Median age, yrs (range)	**33 (21–52)**	**46.5 (23–82)**	43 (29–66)	52 (23–82)
**Sex **				
Female	**2 (12.5)**	**11 (27.5)**	0 (—)	11 (40.7)
Male	**14 (87.5)**	**29 (72.5)**	13 (100)	16 (59.3)
**Race and ethnicity^§^**				
Asian or Pacific Islander	**1 (6.3)**	**1 (2.5)**	1 (7.7)	0 (—)
Black or African American	**5 (31.3)**	**26 (65.0)**	9 (69.2)	17 (63.0)
White	**9 (56.3)**	**7 (17.5)**	1 (7.7)	6 (22.2)
Hispanic or Latino	**1 (6.3)**	**6 (15.0)**	2 (15.4)	4 (14.8)
**MSM status**				
MSM	**11 (68.8)**	**15 (37.5)**	10 (76.9)	5 (18.5)
Non-MSM	**4 (25.0)**	**18 (45.0)**	2 (15.4)	16 (59.3)
Unknown	**1 (6.3)**	**7 (17.5)**	1 (7.7)	6 (22.2)
**HIV status**				
Positive	**9 (56.3)**	**13 (32.5)**	13 (100)	0 (—)
Negative	**7 (43.8)**	**27 (67.5)**	0 (—)	27 (100)
**Previous syphilis diagnosis**				
Yes	**—**	**14 (35.0)**	6 (46.2)	8 (29.6)
No	**—**	**21 (52.5)**	6 (46.2)	15 (55.6)
Unknown	**—**	**5 (12.5)**	1 (7.7)	4 (14.8)
**NOO syphilis treatment received**				
Yes	**—**	**36 (90.0)**	11 (84.6)	25 (92.6)
No	**—**	**4 (10.0)**	2 (15.4)	2 (7.4)
**Admitted to hospital**				
Yes	**—**	**23 (57.5)**	9 (69.2)	14 (51.9)
No	**—**	**5 (12.5)**	0 (—)	5 (18.5)
Unknown	**—**	**12 (30.0)**	4 (30.8)	8 (29.6)
**Sign or symptom^¶^**				
Acute headache	**—**	**9 (22.5)**	5 (38.5)	4 (14.8)
Central nervous system deficits	**—**	**4 (10.0)**	3 (23.1)	1 (3.7)
Decreased vision	**—**	**17 (42.5)**	4 (30.8)	13 (48.1)
Fever	**—**	**3 (7.5)**	2 (15.4)	1 (3.7)
Gait difficulty	**—**	**8 (20.0)**	3 (23.1)	5 (18.5)
Hearing loss	**—**	**2 (5.0)**	0 (—)	2 (7.4)
Lymphadenopathy	**—**	**1 (2.5)**	1 (7.7)	0 (—)
Malaise	**—**	**3 (7.5)**	2 (15.4)	1 (3.7)
Meningismus	**—**	**1 (2.5)**	0 (—)	1 (3.7)
Optic neuritis	**—**	**3 (7.5)**	2 (15.4)	1 (3.7)
Photophobia	**—**	**6 (15.0)**	1 (7.7)	5 (18.5)
Rash	**—**	**10 (25.0)**	2 (15.4)	8 (29.6)
Retinitis	**—**	**6 (15.0)**	2 (15.4)	4 (14.8)
Sensory change	**—**	**5 (12.5)**	1 (7.7)	4 (14.8)
Ulcer or lesion	**—**	**6 (15.0)**	2 (15.4)	4 (14.8)
Uveitis	**—**	**7 (17.5)**	2 (15.4)	5 (18.5)
Weakness	**—**	**7 (17.5)**	4 (30.8)	3 (11.1)
Undetectable HIV viral load**				
Yes	**—**	**NA**	7 (53.8)	NA
No	**—**	**NA**	6 (46.2)	NA
**CD4^††^<200^§§^**				
Yes	**—**	**NA**	4 (30.8)	NA
No	**—**	**NA**	9 (69.2)	NA
**On HIV antiretroviral therapy^¶¶^**				
Yes	**—**	**NA**	8 (61.5)	NA
No	**—**	**NA**	2 (15.4)	NA
Unknown	**—**	**NA**	3 (23.1)	NA

**Source: **Chicago Health Information Management System and Enhanced HIV/AIDS Reporting System.

**Abbreviations:** MSM = men who have sex with men; NA = not applicable; NOO = neuro-, ocular, and otic.

* As of November 6, 2023.

^†^ Data not available for all variables in 2019.

^§^ Persons of Hispanic or Latino (Hispanic) origin might be of any race but are categorized as Hispanic; all racial groups are non-Hispanic.

^¶^ Signs and symptoms are not mutually exclusive.

** Most recent viral load test result in 2023 was <200 HIV RNA copies/mL.

^††^ CD4 is a class of helper T-lymphocytes.

^§§^ Most recent CD4 count in 2023 was <200 cells/μL.

^¶¶^ Documentation of HIV antiretroviral therapy in previous 12 months.

Among 28 (70.0%) persons for whom signs or symptoms were reported, decreased vision (60.7%), rash (35.7%), and acute headache (32.1%) were the most commonly reported. Among 14 (35.0%) persons whose signs and symptoms were consistent with primary or secondary syphilis, eight had abnormal CSF tests. Signs and symptoms of NOO syphilis were similar in persons who did and did not have HIV.

## Preliminary Conclusions and Actions

During January 1–October 31, 2023, NOO syphilis cases in Chicago were identified more frequently in persons who did not have HIV and who were not MSM compared with cases identified in 2019. Clinicians should consider NOO syphilis even in persons who do not have HIV and who are not MSM. Enhanced surveillance efforts to better understand NOO syphilis trends are needed.

## Footnotes

*
https://www.cdc.gov/sti-statistics/annual/index.html

†
https://www.cdc.gov/syphilis/about/index.html

§ 45 C.F.R. part 46, 21 C.F.R. part 56; 42 U.S.C. Sect. 241(d); 5 U.S.C. Sect. 552a; 44 U.S.C. Sect. 3501 et seq.

## References

[1] Lee MA, Aynalem G, Tabidze I, ; CDC. Symptomatic early neurosyphilis among HIV-positive men who have sex with men—four cities, United States, January 2002–June 2004. MMWR Morb Mortal Wkly Rep 2007;56:625–8. 17597693 PMC6818090

[2] Oliver SE, Aubin M, Atwell L, Ocular syphilis—eight jurisdictions, United States, 2014–2015. MMWR Morb Mortal Wkly Rep 2016;65:1185–8. 10.15585/mmwr.mm6543a2 27811837

[3] Woolston S, Cohen SE, Fanfair RN, Lewis SC, Marra CM, Golden MR. Notes from the field: a cluster of ocular syphilis cases—Seattle, Washington and San Francisco, California, 2014–2015. MMWR Morb Mortal Wkly Rep 2015;64:1150–1. 10.15585/mmwr.mm6440a6 26469141 PMC4894317

[4] CDC. Syphilis (*Treponema pallidum*) case definition. Atlanta, GA: US Department of Health and Human Services, CDC; 2018. https://ndc.services.cdc.gov/case-definitions/syphilis-2018/

[5] Cohen SM, Gray KM, Bañez Ocfemia MC, Johnson AS, Hall HI. The status of the National HIV Surveillance System, United States, 2013. Public Health Rep 2014;129:335–41. 10.1177/003335491412900408 24982536 PMC4037459

